# An Elastic Filtering Algorithm with Visual Perception for Vehicle GNSS Navigation and Positioning

**DOI:** 10.3390/s24248019

**Published:** 2024-12-16

**Authors:** Wenzhuo Ma, Zhe Yue, Zengzeng Lian, Kezhao Li, Chenchen Sun, Mengshuo Zhang

**Affiliations:** School of Surveying and Mapping, Henan Polytechnic University, Jiaozuo 454003, China; 212204020075@home.hpu.edu.cn (W.M.); zengzenglian@hpu.edu.cn (Z.L.); kz@hpu.edu.cn (K.L.); sunchenchen@home.hpu.edu.cn (C.S.); 212304020071@home.hpu.edu.cn (M.Z.)

**Keywords:** visual perception, vehicle GNSS navigation and positioning, inter-frame differential optical flow method, elastic filtering

## Abstract

Amidst the backdrop of the profound synergy between navigation and visual perception, there is an urgent demand for accurate real-time vehicle positioning in urban environments. However, the existing global navigation satellite system (GNSS) algorithms based on Kalman filters fall short of precision. In response, we introduce an elastic filtering algorithm with visual perception for vehicle GNSS navigation and positioning. Firstly, the visual perception system captures real-time environmental data around the vehicle. It utilizes the interframe differential optical flow method and vehicle state switching characteristics to assess the current driving status. Secondly, we design an elastic filtering model specifically for various vehicle states. This model enhances the precision of Kalman filter-based GNSS navigation. In urban driving, vehicles often experience frequent stationary parking. To address this, we incorporate a zero-speed constraint to further refine vehicle location data when the vehicle is stationary. This constraint matches the data with the appropriate elastic filtering model. Ultimately, we conduct simulation and real-world vehicle navigation experiments to confirm the validity and rationality of our proposed algorithm. Compared with the conventional algorithm and the existing interactive multi-model algorithm, the proposed algorithm significantly improves the navigation and positioning accuracy of vehicle GNSS in urban environments. Compared to the commonly used constant acceleration (CA) and Constant Velocity (CV) models, there has been a significant improvement in positioning accuracy. Furthermore, when benchmarked against the more advanced interactive multi-model (IMM) model, the method proposed in this paper has enhanced the positioning accuracy enhancements in three dimensions: 21.8%, 20.9%, and 31.3%, respectively.

## 1. Introduction

The relentless march of progress has accelerated the pace of urbanization, thereby increasing the demand for precise vehicle navigation and positioning in urban environments. Consequently, this has become a pivotal area of interest in the field of navigation research [1,2,3]. The Global Navigation Satellite System (GNSS), a satellite-based radio navigation technology, stands out for its ability to provide all-weather, round-the-clock high-precision positioning and timing capabilities on or near the Earth’s surface, provided that the satellite receiver can capture a sufficient number of high-quality GNSS satellite signals. Significantly, the navigation and positioning accuracy of GNSS do not deteriorate over time. Owing to these compelling benefits, GNSS has been extensively integrated into the domain of vehicle navigation [4,5,6].

In the realm of vehicle GNSS navigation and positioning, the Kalman filter, when integrated with vehicle positioning models, is frequently employed to determine a vehicle’s location. For example, the reference introduces a novel joint positioning algorithm incorporating a constant speed model within the onboard GNSS filtering process [7]. Reference showcases an algorithm designed for the self-localization and tracking of autonomous vehicles that harnesses a Kalman filter-based GNSS filtering model predicated on a constant speed model, effectively constraining vehicle position errors [8]. The reference presents a vehicle combination positioning method founded on Bayesian theory, utilizing a GNSS filtering constant acceleration model to achieve high-precision outcomes that align with the needs of intelligent vehicle positioning [9]. Reference proposes a new motion target tracking algorithm that relies on a Kalman filter, with onboard GNSS filtering adopting a constant acceleration model to attain centimeter-level tracking accuracy [10]. Lastly, the reference assesses an optimized motion positioning method that employs a GNSS filtering constant acceleration model, significantly enhancing positioning accuracy and continuity [11]. Both constant speed and constant acceleration models are extensively utilized in vehicle GNSS navigation and positioning based on the Kalman filter. Moreover, the synergistic application of these two models can also substantially enhance the accuracy of vehicle navigation and positioning [12].

Although the constant speed and constant acceleration models used in vehicle-mounted GNSS filtering can significantly enhance the accuracy of navigation and positioning, they are typically employed in isolation or in tandem. In the intricate and variable motion states of a vehicle, such as during turns, these models may not adequately account for non-linear motion and special motion states, potentially leading to inaccuracies in the dynamic positioning process of vehicle motion [13,14]. Furthermore, while it is indisputable that traditional motion models based on the Kalman filter can effectively enhance the accuracy of vehicle GNSS navigation and positioning, they often fall short when capturing the full spectrum of a vehicle’s motion states in real-world environments. This limitation suggests that the models may not fully encapsulate the complex dynamic behaviors exhibited by vehicles in their actual operational contexts. Consequently, the exploration of more adaptable and varied motion models, as well as the optimization and enhancement of existing models, is imperative for advancing the precision of vehicular GNSS navigation and positioning systems [15,16,17].

Recognizing this challenge, scholars have primarily proposed two strategies: developing novel Kalman-based vehicle-mounted GNSS models and refining and enhancing existing models and algorithms [18,19]. In this vein, researchers have proposed and implemented a first-order Gaussian Markov system kinematics model coupled with a multirate-adaptive Kalman filter algorithm. This innovative approach has demonstrated a significant boost in the accuracy of vehicle GNSS navigation and positioning compared to traditional models [20,21]. Furthermore, the development of the Interacting Multiple Model (IMM) Kalman filter has been a notable advancement. This model amalgamates various system motion models for state estimation and synthesizes the outcomes in a weighted manner, thereby enhancing the precision of vehicle GNSS navigation and positioning [22,23]. However, despite these studies achieving notable advancements in enhancing vehicle GNSS navigation positioning accuracy, the complexity and variability of vehicle motion states in real environments means that current models are not always capable of reflecting real-time changes in vehicle motion states, indicating that there is potential for further improvement in positioning accuracy [24,25,26]. At this juncture, incorporating additional sensors to capture the vehicle’s real-time motion state while ensuring the enhancement of real-time performance in vehicle GNSS navigation and positioning becomes a more pragmatic approach, especially when considering the cost implications of such additions to the vehicle. Consequently, the adoption of fisheye cameras, which leverage visual perception to provide abundant and detailed information, has gained widespread acceptance [27,28].

In summary, based on previous efforts, future research should consider complex and dynamic real-time vehicle-mounted GNSS filtering models to adapt to the changing driving environment and achieve a higher level of vehicle-mounted GNSS navigation and positioning accuracy. Therefore, with the help of the camera, a low-cost environmental perception tool that can provide massive information [29], which proposes an elastic filtering algorithm for vehicle GNSS navigation and positioning based on visual perception to solve the problem that the current vehicle GNSS navigation and positioning system cannot obtain the variable motion state of the vehicle in real-time. The algorithm can judge the different motion states of the vehicle in the case of real-time acquisition of the surrounding environment information of the vehicle motion and flexibly match the vehicle motion state model. When the vehicle is stationary, it is further supplemented with zero-speed constraints to improve the positioning accuracy of the vehicle GNSS navigation based on the Kalman filter in an urban environment. The structure of this paper is arranged as follows: the first part is the introduction, which outlines the theoretical background and research status of the proposed method; the second part describes the theoretical content of the constant speed and constant acceleration models based on Kalman filtering for vehicular GNSS; the third part introduces the theory and methods for vehicle state determination; the fourth part presents the experiments and result analysis, including both simulation and real-world experiments, which validate the effectiveness and rationality of the proposed method; and the fifth part concludes the paper by summarizing its advantages and limitations, as well as indicating future research directions and topics.

In conclusion, drawing upon the collective efforts of previous research, future endeavors should focus on developing sophisticated and dynamic real-time vehicle-mounted GNSS filtering models that can adapt to ever-evolving driving conditions, thereby achieving a higher degree of accuracy in vehicle-mounted GNSS navigation and positioning. Consequently, leveraging a camera is a cost-effective environmental perception tool that can gather extensive information [29]. Therefore, this paper introduces an elastic filtering algorithm with visual perception for vehicle GNSS navigation and positioning. This algorithm is designed to address the current limitations of vehicle GNSS navigation and positioning systems in capturing the variable motion states of vehicles in real-time. The proposed algorithm can discern the diverse motion states of a vehicle by acquiring real-time information regarding the vehicle’s surrounding environment. It can then be flexibly aligned with the appropriate vehicle motion state model. When the vehicle is at rest, the algorithm further incorporates zero-speed constraints to enhance the positioning accuracy of vehicle GNSS navigation based on the Kalman filter, particularly in urban environments. The structure of this paper is organized as follows: The initial section serves as an introduction, providing an overview of the theoretical framework and the current state of research of the proposed method. The second section explores the theoretical foundations of the constant-speed and constant-acceleration models, which utilize Kalman filtering in vehicular GNSS systems. The third section elucidates the theory and methodologies for determining the state of the vehicle. The fourth section showcases the experimental setup and the analysis of the results, encompassing both simulated and real-world experiments, which validate the efficacy and rationality of the proposed method. The final section offers a conclusion to the paper by summarizing its strengths and weaknesses, and suggesting potential avenues for future research and exploration.

## 2. Vehicle GNSS Constant Speed and Constant Acceleration Model Based on Kalman Filter

This algorithm primarily encompasses five distinct formulas that are bifurcated into prediction and state update phases, as delineated in Formulas (1)–(5). The quintessential application of this method involves forecasting and refining the three-dimensional coordinates of the position, velocity, and acceleration of the vehicle contingent upon the varying operational conditions of the vehicle. Generally, Kalman filtering encompasses a state prediction module and a measurement update module, following a specific process outlined below:

(a) State prediction module

(1) $$X_{i}=AX_{i-1}$$

(2) $$\hat{P_{i}}=AP_{i-1}A^{T}+Q$$

where $X_{i}$ represents the prior state estimate, A represents the corresponding state transfer matrix according to the different vehicle motion models, $P_{i}$ is the predicted covariance matrix of the state vector, and Q is the corresponding system noise covariance matrix.

(b) Measurement update module

(3) $$K=\hat{P_{i}}H^{T}{\left[HP_{i}H^{T}+R\right]}^{-1}$$

(4) $$X_{i}={\hat{X}}_{i}+K\left[Z_{i}-HX_{i}\right]$$

(5) $$P_{i}=\left[I-KH\right]{\hat{P}}_{i}$$

where H is denoted as the measurement relationship matrix; in this paper, it is used as a direction cosine matrix [30], R is the observation noise covariance matrix, K is the Kalman filter gain, $Z_{i}$ represents the actual observation value, and I is the identity matrix. The GNSS vehicle navigation positioning constant speed and constant acceleration models is mainly reflected in the changes in the state transition matrix A in Kalman filtering. A in the constant speed model is represented as [8]:

(6) $$A=\left[\begin{array}{cccccc} 1 & 0 & 0 & T & 0 & 0\\ 0 & 1 & 0 & 0 & T & 0\\ 0 & 0 & 1 & 0 & 0 & T\\ 0 & 0 & 0 & 1 & 0 & 0\\ 0 & 0 & 0 & 0 & 1 & 0\\ 0 & 0 & 0 & 0 & 0 & 1 \end{array}\right]$$

A in constant acceleration model is represented as [10]:

(7) $$A=\left[\begin{array}{ccccccccc} 1 & 0 & 0 & T & 0 & 0 & T^{2}/2 & 0 & 0\\ 0 & 1 & 0 & 0 & T & 0 & 0 & T^{2}/2 & 0\\ 0 & 0 & 1 & 0 & 0 & T & 0 & 0 & T^{2}/2\\ 0 & 0 & 0 & 1 & 0 & 0 & T & 0 & 0\\ 0 & 0 & 0 & 0 & 1 & 0 & 0 & T & 0\\ 0 & 0 & 0 & 0 & 0 & 1 & 0 & 0 & T\\ 0 & 0 & 0 & 0 & 0 & 0 & 1 & 0 & 0\\ 0 & 0 & 0 & 0 & 0 & 0 & 0 & 1 & 0\\ 0 & 0 & 0 & 0 & 0 & 0 & 0 & 0 & 1 \end{array}\right]$$

where T represents the time interval.

By establishing and applying constant speed and constant acceleration models, the positioning accuracy of vehicle GNSS navigation can be significantly enhanced. However, within an intricate urban environment, the fluctuating motion states of vehicles render these models inadequate for accommodating the diverse positioning states required, thereby underscoring the pressing need for improved positioning accuracy. Moreover, an interactive multi-model approach has been developed by leveraging the state estimation of the Kalman filter model to fuse the results in a weighted manner. This technique, when juxtaposed with the constant speed and constant acceleration models, demonstrates superior positioning accuracy. This advancement is pivotal in addressing the challenges posed by the dynamic urban landscape, where vehicles frequently transition between different motion states, thus necessitating a more sophisticated model to ensure precise and reliable positioning.

## 3. The Proposed Method

As illustrated in the second part, when filtering GNSS data in vehicles, Constant Acceleration (CA), Constant Velocity (CV), and Constant Deceleration (CD) are filtering models with different parameter contents in the prediction and update equations. The IMM is a weighted combination of the CA and CV models [23]. However, these models are unable to meet the demands of vehicles that frequently change their motion states within a short period. They also fail to determine the vehicle’s state in real time and design flexible constraint models to enhance the vehicle positioning accuracy.

**Figure 1:**
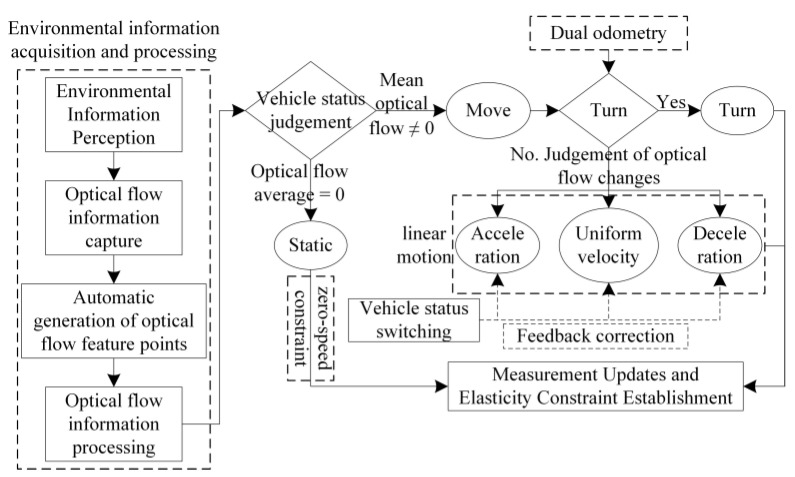
Flowchart of the Proposed Algorithm.

In this study, a sky fisheye camera is employed to capture the surrounding environmental data, and the inter-frame differential optical flow method is utilized to derive the mean value of the optical flow feature points, thereby precisely discerning the motion and static states of the vehicle. During vehicular movement, the turning state is identified using a differential meter, which then prompts the design of the corresponding elastic filtering model. Subsequently, based on the mean value of the optical flow feature points obtained, the intervals of constant speed, constant acceleration, and constant deceleration vehicle motion are identified, catering to the linear motion of the vehicle post-turning. Feedback correction is applied in conjunction with vehicle state transitions, such as coming to a halt after deceleration or resuming motion and acceleration, to dynamically construct a vehicle GNSS navigation and positioning constraint model that is elastic and adaptable to various vehicle motion states. In scenarios where the vehicle is stationary, which is a common occurrence in urban parking environments, this paper develops a zero-speed constraint model. This model is integrated into a flexible onboard GNSS navigation and positioning algorithm to ascertain when the vehicle is in a stationary state, imposing strict constraints on vehicle positioning to enhance accuracy during such periods. Ultimately, a comparative analysis between simulation and real-world experiments is conducted to substantiate the efficacy of the proposed model in enhancing navigation and positioning accuracy. A flowchart of the algorithm introduced in this paper is depicted in Figure 1.

### 3.1. Vehicle State Assessment Theory and Methods

#### 3.1.1. Introduction to Experimental Equipment

**Figure 2:**
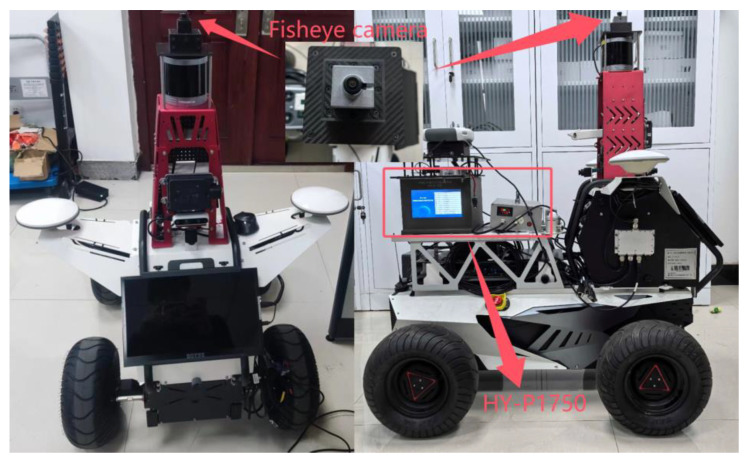
Experimental Platform.

The visual perception tool employed in this experiment is an upward-facing fisheye camera, which is affixed to the seamless integrated navigation and positioning platform of Henan Polytechnic University. This camera is distinguished by its affordability and capacity to capture vast amounts of environmental information. Operating on the Ubuntu system, it excels in information collection and development, supporting image information collection at a rate of 20 frames per second. This capability allows it to efficiently acquire and analyze the surrounding state in a dynamic environment, thus offering real-time support for vehicle navigation and positioning. The experimental platform is illustrated in Figure 2.

This article employs a fisheye camera to collect image data, while the HY-P1750 high-precision integrated navigation system and Inertial Explorer 9.0 software are used to provide the reference truth, the equipment is provided by Hua Yuan Xing Tong Technology Co., Ltd., located in Beijing, China. The reference device provides high-precision true knowledge information, a dual-frequency RTK accuracy of 2 cm, and a post-processing accuracy of 5 mm.

Among them, the parameters of the experimental equipment are shown in Table 1.

The equipment is provided by Dao Fei Technology Co., Ltd., located in Qingdao, China. The experimental equipment parameters demonstrated in the aforementioned experiment indicate that the device is capable of meeting the experimental requirements. By using this camera to collect environmental information about the vehicle’s travel, we can obtain information that is conducive to improving the accuracy of vehicular GNSS navigation and positioning. This, in turn, verifies the objective of this paper, which is to rely on external low-cost visual sensors to enhance the accuracy of vehicular navigation and positioning in urban environments.

**Figure 3:**
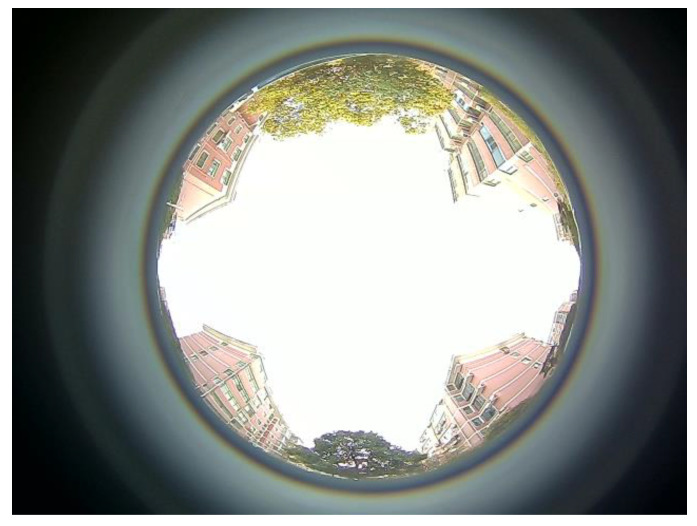
Captured Upward-Facing Environmental Information.

Utilizing this experimental platform, data are harvested from the upward-facing fisheye camera (operating on the Ubuntu system) to form ROS data packets. The designed program then processes these packets to obtain the environmental information surrounding the test vehicle’s travel. These upward-facing environmental images are highly compatible with urban settings and provide a reliable data foundation for subsequent navigation, positioning, and dynamic analysis. Notably, the image content captures the intricate surroundings of the vehicle, which serves to augment the system’s intelligent perception capabilities. These images are instrumental in providing a detailed and accurate representation of the vehicle environment, which is crucial for advanced navigation and positioning systems. The captured images are displayed in Figure 3.

#### 3.1.2. Inter-Frame Differential Optical Flow Method

This methodology entails assigning a velocity vector to each pixel point within the image, thereby creating an image motion field. At any given moment of motion, each point in the image is mapped to a distinct point on the three-dimensional object. This mapping is established through the projection relationship, which enables dynamic image analysis based on the velocity vector attributes of the individual pixel points. The technique employed in this study is the Lucas-Kanade (LK) optical flow method.

Suppose that the time of the previous frame is t and the time of the next frame is t+δt. Then the position of the pixel point I(x,y,z,t) of the previous frame i in the later frame is denoted as

I(x+δx,y+δy,z+δz,t+δt)

where x, y, and z are the position components of the three directions; δ is the amount of change; and t is the previous frame time.

Based on the assumption of brightness constancy, the pixel point change formula between frames can be expressed as follows:

(8) I(x,y,z,t)=I(x+δx,y+δy,z+δz,t+δt)

where ∂ is the differential sign.

Expanding the right-hand side of Equation (6) using a Taylor series based on the assumption of small motion yields the following expression:

(9) $$I(x+\delta x,y+\delta y,z+\delta z,t+\delta t)=I(x,y,z,t)+\frac{\partial I}{\partial x}\delta x+\frac{\partial I}{\partial y}\delta y+\frac{\partial I}{\partial z}\delta z+\frac{\partial I}{\partial t}\delta t+HL$$

where HL is a higher-order term in the Taylor series expansion, that can be ignored in the case of small movement.

Combining Formulas (6) and (7), the variations in the formulas can be expressed as follows:

(10) $$\frac{\partial I}{\partial x}\delta x+\frac{\partial I}{\partial y}\delta y+\frac{\partial I}{\partial z}\delta z+\frac{\partial I}{\partial t}\delta t=0$$

Since this experiment involves two-dimensional skyscape information images captured by a skyward camera, it is sufficient to consider only x,y,t. Let Ix,Iy,It represent the difference of the image in the (x,y,t) respectively. It can be expressed as $I_{x}V_{x}+I_{y}V_{y}=-I_{t}$, where $I_{x}$ represents the rate of change of pixel image pixel in the x direction, $V_{x}$ is the vector of the optical flow in the x direction, $I_{y}$ represents the position of the pixel in the y direction, $V_{y}$ denotes the vector of the optical flow in the y direction, and $I_{t}$ represents the rate of change of the image with time t. Additionally, with the inclusion of the spatial consistency assumption, the Lucas-Kanade algorithm constructs nine formulas using nine-pixel points within a 3 × 3 window. It is abbreviated as:

(11) $$\left[\begin{array}{cc} I_{x1} & I_{y1}\\ I_{x2} & I_{y2}\\ \vdots & \vdots \\ I_{x9} & I_{y9} \end{array}\right]\left[\begin{array}{c} V_{x}\\ V_{y} \end{array}\right]=\left[\begin{array}{c} -I_{t1}\\ -I_{t2}\\ \vdots \\ -I_{t9} \end{array}\right]$$

This formula can be rewritten in the following vector form:

(12) $$A_{G}\vec{v}=-b$$

where $A_{G}$ is the set of pixels, $\vec{v}$ represents the optical flow, and b is the moment corresponding to the optical flow.

In Equation (10), both sides of the formula correspond to each other, with the right side of the formula set −b to isolate the positive and negative signs from the value. The least squares method is then employed to solve for the optical flow vectors.

(13) $$A_{G}^{T}A_{G}\vec{v}=A_{G}^{T}(-b)$$

(14) $$\vec{v}={\left(A_{G}^{T}A_{G}\right)}^{-1}A_{G}^{T}(-b)$$

where T is the transposed symbol.

Equation (9) can also be expressed in matrix form as follows:

(15) $$\left[\begin{array}{c} V_{x}\\ V_{y} \end{array}\right]={\left[\begin{array}{cc} \sum I_{xi}^{2} & \sum I_{xi}I_{yi}\\ \sum I_{xi}I_{yi} & \sum I_{yi}^{2} \end{array}\right]}^{-1}\left[\begin{array}{c} -\sum I_{xi}I_{ti}\\ -\sum I_{yi}I_{ti} \end{array}\right]$$

where $I_{xi}$ and $I_{yi}$ are the i-th pixel at $I_{ti}$ and the corresponding moments.

Equation (13) indicates that by accumulating the partial derivatives of the neighborhood pixel point in three dimensions for matrix operation, the optical flow $\left(V_{x},V_{y}\right)$ of the point can be calculated.

#### 3.1.3. Vehicle State Assessment Method

**Figure 4:**
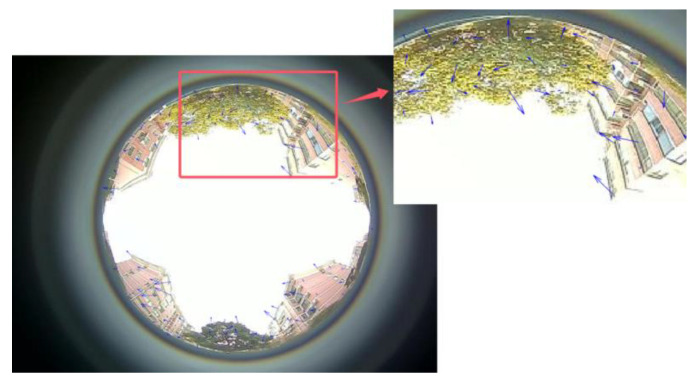
Inter-Frame Differential Optical Flow Visualization.

Initially, a visual perception tool is employed to capture the driving environment surrounding the vehicle. Utilizing the principles outlined in Formulas (8)–(15), optical flow information is extracted through the frame difference optical flow method. This process leads to automatically generating optical flow feature points, as shown in the enlarged view of Figure 4 with the blue line.

**Figure 5:**
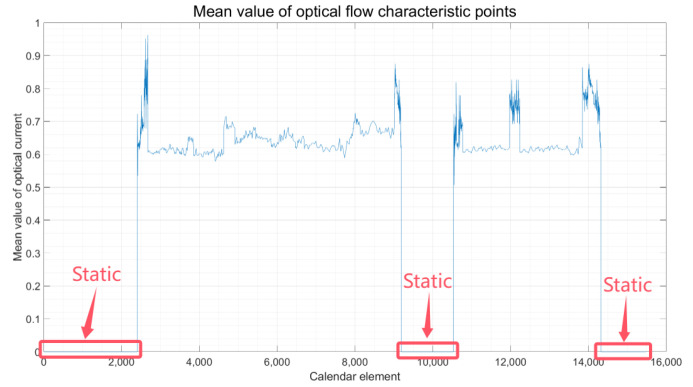
Vehicle Stationary Interval Result.

Subsequently, the optical flow information is extracted, and its values are averaged. By analyzing the mean value of the optical flow feature points, the intervals of motion and the rest of the vehicle can be pinpointed with precision. At this time, the results of the mean value of the optical flow feature points and the judgment of the vehicle state for the different motion intervals of the vehicle detected by this method are shown in Figure 5.

The judgment steps for the different motion state intervals of the vehicle are shown below.

**Figure 6:**
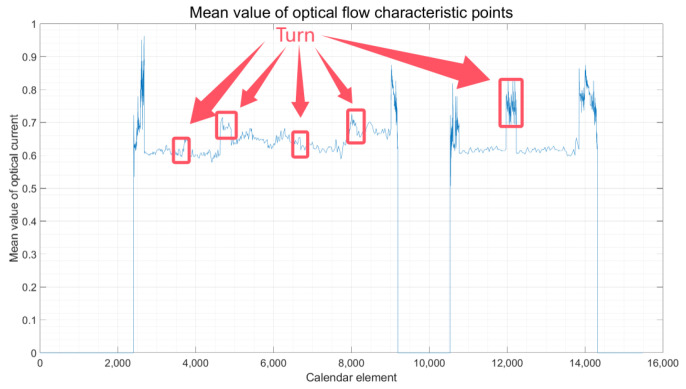
Vehicle Turning Interval Result.

**Figure 7:**
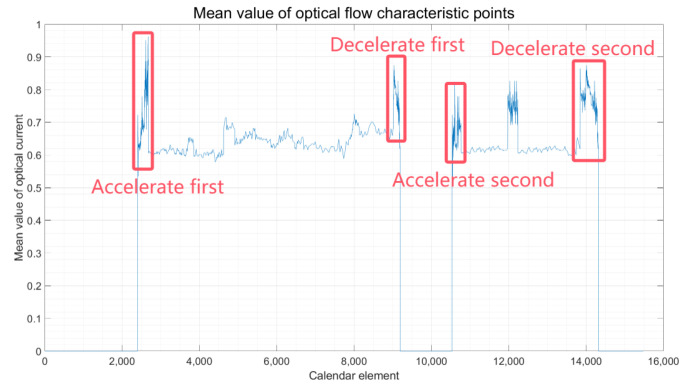
Vehicle Acceleration and Deceleration Interval.

①. Judgment of Motion and Stationary State: When the vehicle is at rest, the images captured by the visual perception tool are presented in Figure 3. In this state, due to the lack of change in the imagery, establishing a video observation from these static images will yield no optical flow feature points, as illustrated in Figure 5, where the average optical flow is null. Conversely, when the vehicle is in motion, the images within the video sequence exhibit temporal changes, resulting in detectable variations in the optical flow. As demonstrated in Figure 4, this scenario corresponds to a case in which the average optical flow is non-zero, signifying the vehicle’s movement. Thus, by assessing the presence or absence of the mean value of the optical flow feature points, the motion or stillness of a vehicle can be determined with high accuracy. By visually inspecting the displayed images, the experiment can be observed to be static before commencement, dynamic during the process, and static again upon conclusion. This visual assessment corroborates the vehicle’s state transitions throughout the experiment.

②. Judgment of Turning State: Drawing from the intervals of vehicle motion and stationary states identified in step ①, when the vehicle is executing a turn, the average optical flow in the captured images might not exhibit a significant deviation from that of linear motion, or it might undergo abrupt changes in certain instances. This makes it challenging to ascertain the vehicle’s turning state based solely on the optical flow average. To address this problem, the experimental platform, as depicted in Figure 2, is equipped with a differential meter. When a turn is detected, the vehicle’s state can be accurately determined to be turning by analyzing the disparity between the two wheel speeds. The outcomes of this assessment are presented in Figure 6.

③. Judgment of constant speed, constant acceleration, and constant deceleration states in straight-line motion: Building upon the identification of the vehicle’s stationary state through step ① and the determination of the vehicle’s turning state through step ②, with the implementation of high-priority judgments designed to ascertain these states, as illustrated in the experimental flowchart in Figure 1, by scrutinizing the regular fluctuations in the average optical flow feature points over a brief timeframe, the intervals during which the vehicle is undergoing acceleration and deceleration can be precisely determined, as depicted in Figure 7.

When the vehicle is undergoing constant acceleration, the average optical flow threshold is observed to rise over a brief period, as evidenced in the “Accelerate first” section of the figure mentioned above. It is noticeable that during this interval, the mean value of the optical flow feature points exhibits an upward trend. On the flip side, when the vehicle is in a state of constant deceleration, the average optical flow threshold similarly tends to increase over a short period, as highlighted in the “Decelerate first” section of Figure 7. Within this interval, the average optical flow feature points display a downward trend, enabling the identification of the corresponding vehicle motion state. Specifically, acceleration is typically observed at the onset of movement from a stationary state, while deceleration is noted as the vehicle approaches a halt. The feedback correction mechanism ascertains the vehicle’s constant deceleration and constant acceleration states, thus achieving a precise identification of the vehicle’s intervals between constant acceleration and constant deceleration. Based on the typical state transition characteristics of the vehicle—acceleration upon starting from rest and deceleration before stopping—the vehicle’s motion states are accurately distinguished and judged. Under the determinations made in steps ① and ②, and this segment, once the vehicle’s static, constant acceleration, constant deceleration, and turning states have been adjudged, the intervals with known state results are excluded. The remaining intervals are identified as times when the vehicle is in a constant speed motion state. This process facilitates the accurate differentiation and judgment of the vehicle’s motion state.

The statistics of the different motion intervals of the vehicle in the actual experiment are shown in Table 2 below.

Combining the results from Figure 5 regarding the mean optical flow with the intervals of different motion states of the vehicle, as identified in Table 1, it is observed that when the vehicle is stationary, the mean optical flow exhibits a significant change, leading to the highest accuracy rate in judgment. The pronounced variation in the mean optical flow provides a clear indication of the vehicle’s state due to its significant fluctuation. Following closely, the vehicle, during a turn, achieves an accuracy rate of 97%. This is facilitated by the differential speedometer, which aids in judgment. In contrast, a vehicle engaged in normal straight-line travel exhibits varying acceleration and deceleration speeds due to changes in motion velocity. Consequently, the judgment accuracy in this scenario is approximately 92%. This observation underscores the need for future work to build upon the methodology presented in this paper with the aim of enhancing the judgment accuracy for vehicles in straight-line motion.

### 3.2. Zero-Speed Constraint

Utilizing the vehicle state judgment step ① to identify the static intervals of the vehicle and considering the frequent occurrence of stationary states in urban environments, a vehicle zero-speed constraint model is established. This model leverages the visual perception tool to accurately determine the vehicle’s static intervals by analyzing the mean value of the inter-frame differential optical flow feature points. By incorporating this model, the vehicle’s motion in three dimensions can be effectively constrained in terms of speed. The accuracy of the vehicle’s three-dimensional positioning can be enhanced by integrating this model with an elastic constraint model. The detailed implementation is as follows.

(16) $$V=\left[\begin{array}{c} V_{x}\\ V_{y}\\ V_{z} \end{array}\right]\approx \left[\begin{array}{c} 0\\ 0\\ 0 \end{array}\right]$$

Correspondingly, when the vehicle is stationary, its state transfer matrix A will change accordingly.

(17) $$A=\left[\begin{array}{ccc} 1 & 0 & 0\\ 0 & 1 & 0\\ 0 & 0 & 1 \end{array}\right]$$

The rationale behind designing this model to assist vehicular GNSS navigation and positioning is primarily due to the frequent stationary states of vehicles in urban environments, such as roadside parking and waiting at red lights. When the vehicle is stationary, by constraining its velocity in three directions and integrating the corresponding Kalman filter model for stationary vehicles, we can achieve the goal of high-precision navigation and positioning.

### 3.3. Multi-Mode Elastic Constraints

Utilizing the precise vehicle motion data derived from the vehicle state assessment, a vehicle-mounted GNSS navigation and positioning system employing elastic filtering has been crafted to align with the determined operational state. This system is anchored on the Kalman prediction and update formulas, with a tailored state transition matrix for various operational modes. Furthermore, the incorporation of a zero-speed constraint enhances the accuracy of the vehicle navigation system’s positioning capabilities.

In scenarios where the vehicle is determined to be stationary, an elastic model and a zero-speed constraint are meticulously crafted using Formulas (1)–(5), (16) and (17). For instances when the vehicle is identified as moving at a constant velocity, the corresponding elastic model is developed utilizing Formulas (1)–(6). In cases of constant acceleration, the elastic model is formulated with Formulas (1)–(5) and (7). For vehicles undergoing constant deceleration, the state transition matrix and correction of the elastic model are designed in a manner similar to the constant acceleration model. During vehicle turns, the Constant Turn Rate and Velocity (CTRV) model is implemented, which is defined by the following expression [31]:

(18) $$x_{turn}=\left[\begin{array}{ccccc} x_{turn} & y_{turn} & v_{turn} & \theta_{turn} & \omega_{turn} \end{array}\right]$$

where $\left(x_{turn},y_{turn}\right)$ is the position of the vehicle, $v_{turn}$ is the magnitude of the velocity of the vehicle in the traveling direction, $\theta_{turn}$ represents the angle of deflection between the velocity vector of the vehicle and the horizontal axis, and $\omega_{turn}$ represents the rate of change of deflection.

Meanwhile, the state transition formula can be expressed as follows:

(19) $$x_{turn}\left(t+\Delta t\right)=\Delta f\left(x_{turn}\left(t\right)\right)+x_{turn}\left(t\right)$$

where Δt represents the time interval and $\Delta f\left(x_{turn}\left(t\right)\right)$ is the state transfer formula. In the present study, the time interval was set to 0.05 s. The state transition formula is represented as:

(20) $$\Delta f\left(x_{turn}\left(t\right)\right)=\left[\begin{array}{c} \frac{v_{turn}}{\omega_{turn}}\left(\sin\left(\theta_{turn}+\omega_{turn}\Delta t\right)-\sin\left(\theta_{turn}\right)\right)\\ \frac{v_{turn}}{\omega_{turn}}\left(-\cos\left(\theta_{turn}+\omega_{turn}\Delta t\right)+\cos\left(\theta_{turn}\right)\right)\\ 0\\ \omega_{turn}\Delta t\\ 0 \end{array}\right]$$

By leveraging the identified diverse driving states of the vehicle, in conjunction with the zero-speed constraint and a multi-mode elastic filtering model, we have successfully validated the enhanced vehicle navigation positioning accuracy method through experimental verification and analysis. The algorithm proposed is outlined as follows (Algorithm 1).
**Algorithm 1:** An Elastic Filtering Algorithm with Visual Perception for Vehicle GNSS Navigation and Positioning**Begin** (1)Environmental Information Image Perception Acquisition.(2)Frame difference method for capturing optical flow information.(3)Automatic generation of optical flow feature points using formulas (8)–(15).(4)Mean processing of optical flow feature points.
 **Predictive module**
  **If** vehicle stationary   Establish an elastic constraint model using Formulas (1), (2) and (17).  **Else if** vehicle motion   **if** vehicle turning   Establish an elastic constraint model using Formulas (18)–(20). Establish a zero-speed constraint model using Formulas (16) and (17).   **else if** vehicle acceleration    Establish an elastic constraint model using Formulas (1), (2) and (6).   **else if** vehicle constant    Establish an elastic constraint model using Formulas (1), (2) and (7).  **else if** vehicle deceleration   Establish an elastic constraint model using Formulas (1) and (2) and modifying the parameters of Formula (7) to be negative.  **end** **End**  **Update the module**  Design state update using Formulas (3)–(5). (5)Correct the obtained results based on vehicle state switching feedback. **End**

## 4. Experimental Verification and Analysis

As described in Algorithm 1, our approach to in-vehicle GNSS navigation and positioning employs an elastic filtering algorithm complemented by a constant deceleration model to account for vehicular motion. This segment of our study juxtaposes the Kalman filtering applied to the constant speed, constant acceleration, and constant deceleration models of in-vehicle GNSS with existing interactive multi-model algorithms. The purpose of this comparison is to investigate and analyze the varying impacts of these algorithms on the precision of in-vehicle GNSS navigation and positioning. By systematically evaluating these models across various environments and motion states, we can gain a profound understanding of the strengths and limitations of each algorithm. This insight is crucial for offering guidance and recommendations to enhance navigation and positioning accuracy. By conducting comparative analyses of both simulation and real-world experiments, the rationality and efficacy of the elastic constraint algorithm in handling dynamic scenarios can be established.

### 4.1. Simulation Experiment

**Figure 8:**
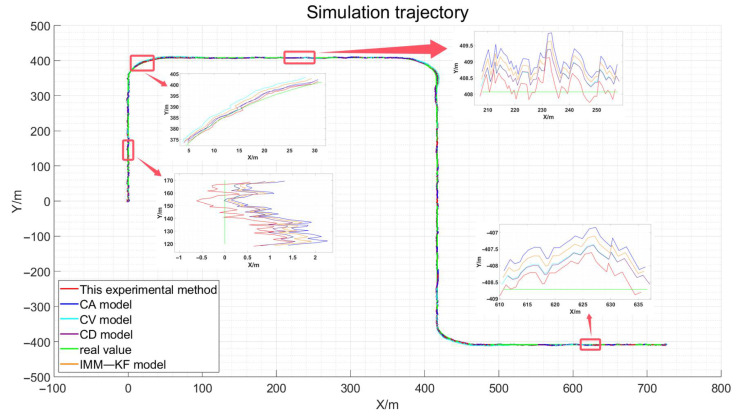
Simulation Experiment Path.

The simulation experiments for the known vehicle motion states across various periods are meticulously designed using MATLAB R2023a, thereby achieving theoretical validation of the proposed algorithm. This simulation replicates a vehicle’s driving trajectory spanning approximately 2000 m, encompassing all motion states observed in the actual experiments, including the stationary, linear motion, and turning phases. By integrating the known vehicle motion states with the elastic Kalman filter constraint model and the zero-speed constraint model, as described in this paper, the theoretical foundation of the experiment is substantiated. The resulting simulated vehicle path from the experiment is depicted in Figure 8.

The path design is meticulously executed through simulation experiments, yielding the intervals of various vehicle states, as detailed in Table 3. In conjunction with the simulation experiment path diagram presented in Figure 8, in the figures presented in this paper, the red line represents the method proposed in this study. CA stands for the Constant Acceleration model, CV for the Constant Velocity model, CD for the Constant Deceleration model, and IMM for the Interacting Multiple Model. it is evident that the depicted path and motion states align with the driving conditions of a vehicle in an urban environment, as they would be in a real-world scenario. The purpose of this simulation experiment is to assess the performance of the proposed model across different motion states in a simulated format, thereby providing theoretical underpinnings for the design of the actual experiment.

**Figure 9:**
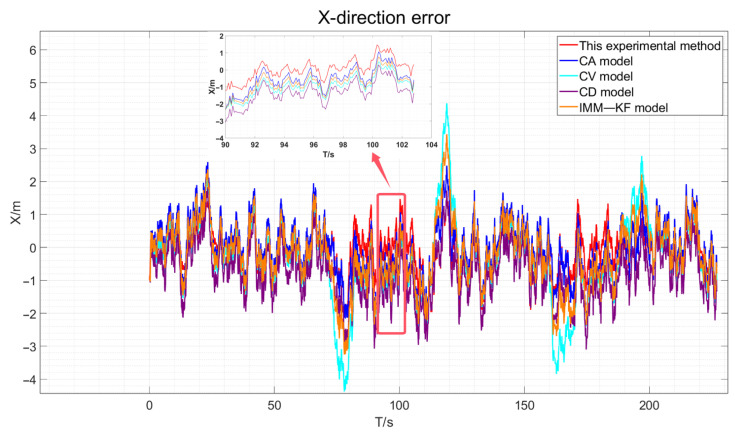
Simulation Experiment X-Direction Positioning Error.

**Figure 10:**
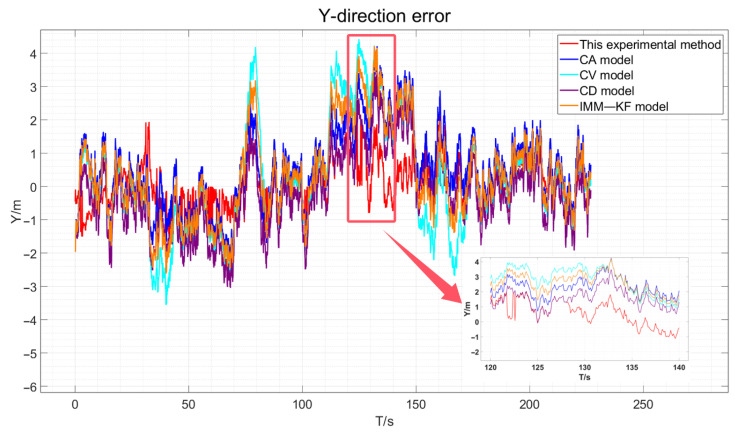
Simulation Experiment Y-Direction Positioning Error.

**Figure 11:**
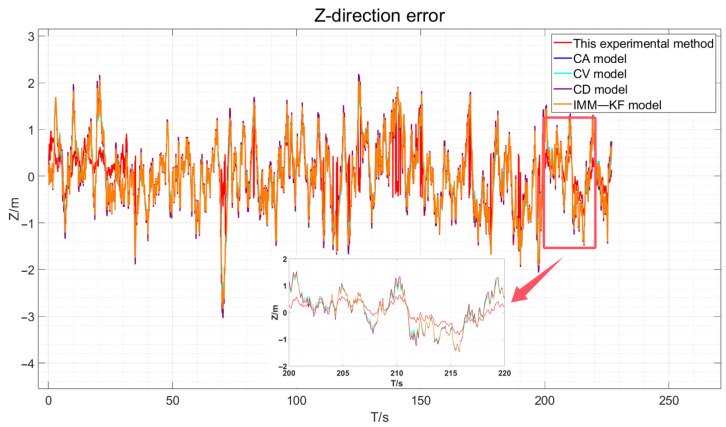
Simulation Experiment Z-Direction Positioning Error.

Furthermore, this figure provides a slice-by-slice amplification of the driving interval for the vehicle segment in our simulation. It can be observed that the red line, representing the method proposed in this paper, shows the highest degree of closeness to the green line compared to the other methods, thereby demonstrating the effectiveness of the approach presented here. However, the advantages of our method are not immediately apparent from the partial interval amplification alone. Given that the figure is a three-dimensional representation, we have conducted a statistical analysis and graphical depiction of the errors in the three-dimensional path. For a more detailed view, please refer to Figure 9, Figure 10 and Figure 11.

Incorporating the results obtained from the simulation experiments as described in this paper, we match the elastic filtering model and augment it with zero-velocity constraints when the vehicle is stationary. We then compare this approach with the Kalman filter-based constant velocity model, constant acceleration model, constant deceleration model, and existing IMM model algorithms for vehicle GNSS navigation and positioning accuracy. This experiment utilizes four distinct models to contrast the simulation experimental true values, presenting the GNSS positioning error results in three dimensions for the vehicle. For more details, please refer to Figure 9, Figure 10 and Figure 11.

By visually analyzing the positioning error results depicted in Figure 9, Figure 10 and Figure 11, particularly by zooming in on the vehicle segments of the designed simulation experiments for comparison, it can be observed that the method proposed in this paper improves the vehicle navigation and positioning accuracy compared to the existing constant velocity, constant acceleration, constant deceleration, and interactive multi-model GNSS filtering processes. The results specifically demonstrate an enhancement in the positioning accuracy over the three models. The statistical analysis of the mean error (ME) and root mean square error (RMSE) in three dimensions is presented in Table 4.

**Figure 12:**
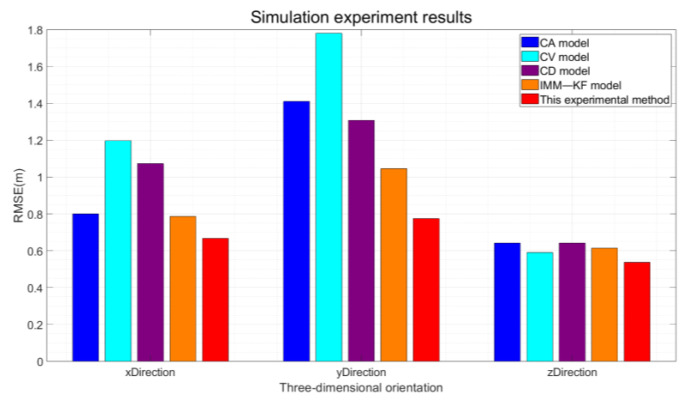
RMSE Statistics in Simulation Experiments.

Based on the results in Table 4, the ME and RMSE of the four models are significantly reduced compared to the positioning results obtained directly through the least squares method. The IMM model shows a superior ME in the Z-direction compared to the method proposed in this paper. However, the larger RMSE is attributed to the fact that while the errors are substantial, they tend to cancel each other out to a greater extent. Notably, the RMSE of the constant velocity, constant acceleration, and constant deceleration models for vehicle GNSS filtering outperforms the least squares results. This is due to the fact that Kalman filter-based algorithms for vehicle GNSS navigation and positioning have been proven effective and are widely used to improve positioning accuracy in diverse settings. The IMM model, which integrates the strengths of the constant velocity and constant acceleration models, enhances the adaptability to vehicle motion by blending the outcomes of Kalman filter state estimation in specific ratios. As a result, the positioning precision is improved compared to the use of either model in isolation. Through simulation experiments, it has been demonstrated that the proposed method can dynamically constrain the positioning accuracy of the vehicle under various motion states, outperforming the IMM algorithm in terms of flexibility. Compared with the four models, it demonstrates a higher degree of alignment, precision, and adaptability. Consequently, the lowest RMSE in the three dimensions corresponds to the highest positioning accuracy. Furthermore, the distinct advantages of the various algorithms are vividly highlighted, and the three-dimensional RMSE results for the four models are quantified. The outcomes are graphically represented in Figure 12.

Figure 12 clearly illustrates that the method proposed in this experiment boasts the smallest RMSE. As corroborated by the simulation results presented in Table 2, the RMSE of the proposed method is substantially reduced when juxtaposed with the constant velocity and acceleration models. The specific enhancements in positioning accuracy are delineated as follows: the proposed method achieves an improvement in positioning accuracy of 16.2%, 45%, and 15.5%in three dimensions, respectively, over the GNSS filtering constant acceleration model; by 44.1%, 56.4%, and 8.6%, respectively, over the constant velocity model; and by 37.6%, 41.1%, and 15.6%, respectively, over the constant deceleration filtering model. Compared to the IMM, the proposed method enhances the accuracy by 14.8%, 25.8%, and 12.5% in the three directions, respectively. This theoretical validation confirms that the elastic filtering algorithm introduced in this paper can effectively enhance the positioning accuracy of vehicle-mounted GNSS navigation systems.

### 4.2. Actual Experiment

To substantiate the theoretical validity of the algorithm presented in this paper, simulation experiments were conducted. Subsequently, field experiments were designed using the skyward environmental data collected at the south campus of Henan Polytechnic University to validate the practical efficacy of the proposed algorithm. As depicted in Figure 2, the experimental vehicle is outfitted with a DW800 wide-angle fisheye camera. This camera boasts a 150° horizontal field of view, continuously gathering skyward information and delivering stable video imagery, even under bright and sunny weather conditions.

#### 4.2.1. Data Collection for Experiments

**Figure 13:**
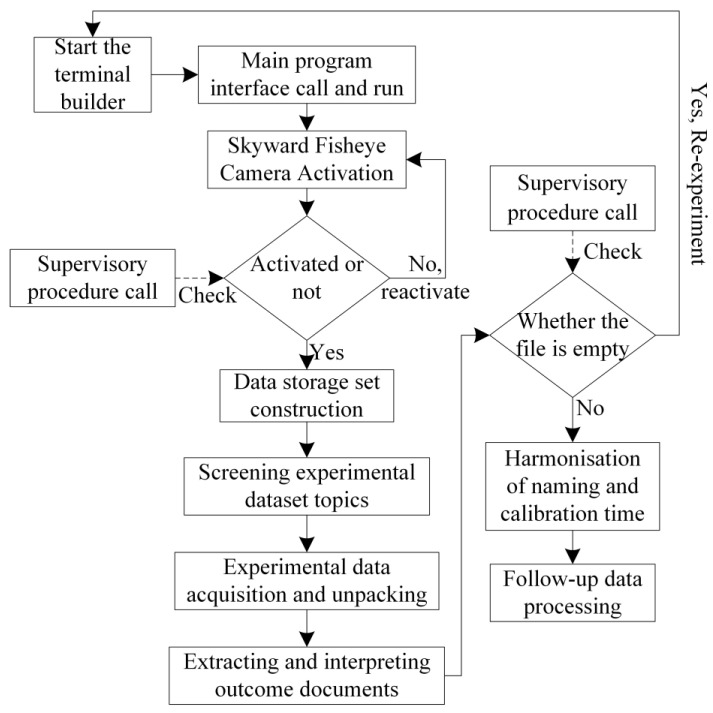
Environmental Information Collection Process.

Utilizing the visual perception tool carrier, specifically the sky fisheye camera, as illustrated in Figure 2, sky information is gathered by constructing an experimental platform for real-world testing. The process of environmental information collection is visually represented in Figure 13 below.

**Figure 14:**
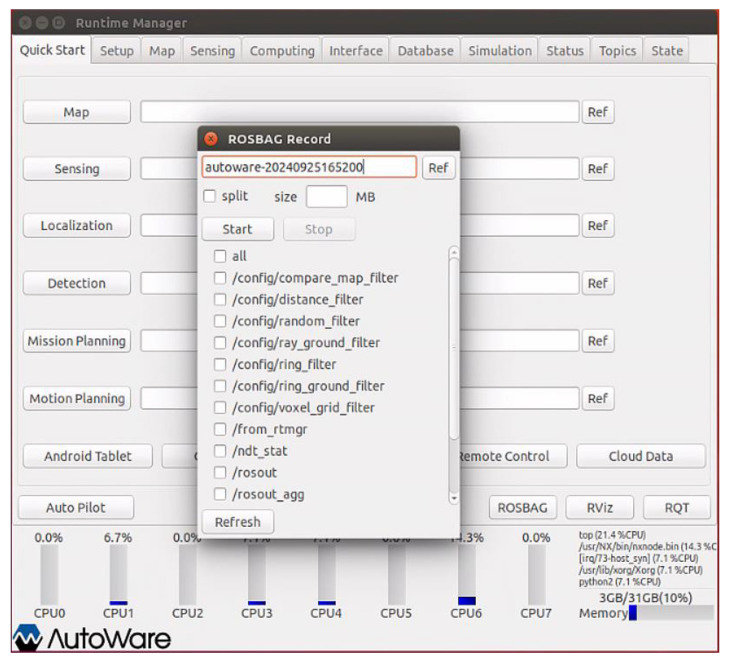
Data Storage Set Construction.

The experimental data acquisition platform is established by leveraging the Ubuntu operating system with the support of NVIDIA graphics. Initiate the terminal to launch the construction program and execute the main program; subsequently, invoke the program to activate the skyward fisheye camera. Activate the supervisory program to ascertain whether the skyward fisheye camera has been successfully activated—if not, reinitiate the program to attempt activation again. Once the skyward fisheye camera is operational, proceed to construct the data storage set for the requisite experimental data collection information, and filter the topic of the experimental data set. The interface for data storage and construction is depicted in Figure 14.

**Figure 15:**
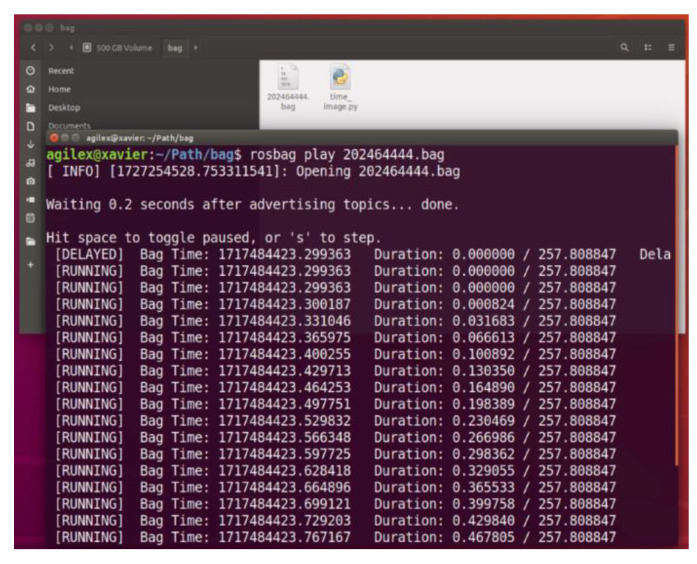
Data File Check Interface.

We collected data using the Autoware 1.1 version. As depicted in Figure 14, experimental data collection can be initiated after configuring the path, topic label, naming conventions, and other pertinent details regarding the image to be collected. Upon completion of data collection, the acquired data file must be decompressed using its designated program, which entails calculating the collected bag file package. The environmental information image is named according to the GNSS time standard format and saved in the PNG format. Once the result file has been extracted and processed, the corresponding supervisory program is invoked to inspect the file. Should the file be devoid of data, the experiment is to be repeated. If the file contains data, the supervisory program proceeds to verify the data file interface, as illustrated in Figure 15.

Upon the validation of the obtained data file, as indicated in Figure 15, proceed to export the data file and organize it into batches, assigning it a consistent naming convention. Convert the timestamps to Coordinated UNIX and perform necessary calibrations to ensure that the experimental data is accurately extracted and ready for analysis.

#### 4.2.2. Actual Experiment Processing

**Figure 16:**
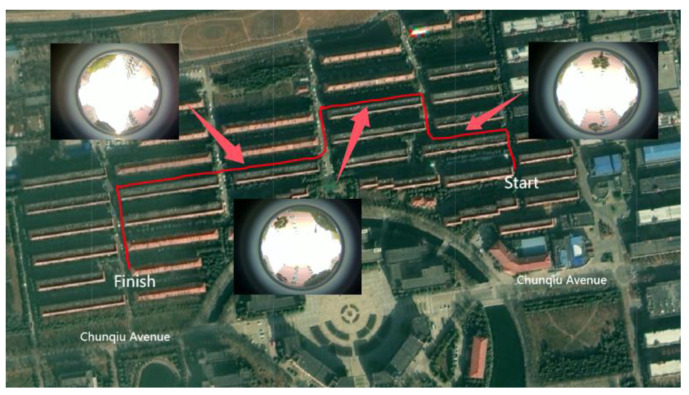
Top view of the experimental path.

Utilizing the experimental environment information collection process depicted in Figure 13, the celestial environmental data is gathered in the vicinity of the southern campus of Henan Polytechnic University, as visualized in Figure 3. This environment facilitates the vehicle’s normal operation in states such as static, linear motion, and turning, with the experimental path extending to a total length of 1000 m. The actual path results obtained from the experiment are shown in Figure 16.

Following the algorithm outlined in Figure 1, which details the vehicle state judgment process, and by examining the optical flow display results presented in Figure 4, as well as the experimental data analysis and judgment outcomes illustrated in Figure 5, Figure 6 and Figure 7, the findings are integrated with the vehicle judgment theory and algorithms proposed in the third section of this paper.

**Figure 17:**
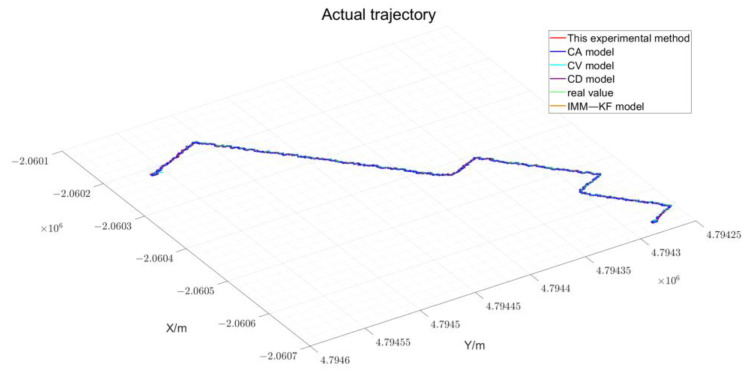
Actual Experimental Path Result.

Based on the statistical intervals of different driving states of the vehicle, the interval results of the judgment are summarized in Table 2, as detailed below. As delineated in Table 2, the experiment encompasses various vehicle motion states. The vehicle is maintained in a stationary state at the commencement, during intervals, and after the experiment. Transitions from a stationary state to acceleration mark the beginning of the vehicle’s movement, with deceleration being a standard phase before parking. Notably, the experiment features numerous intervals of constant vehicle speed, aligning with typical driving conditions. Furthermore, the vehicle executes five turns during the experiment, mirroring the layout of urban streets, which underscores the rationality and effectiveness of the collected experimental data. To gain a comprehensive understanding of the results presented in Table 3 and to visually corroborate the experimental outcomes, the paths derived from the actual experiments have been charted, with the findings graphically represented in Figure 17.

**Figure 18:**
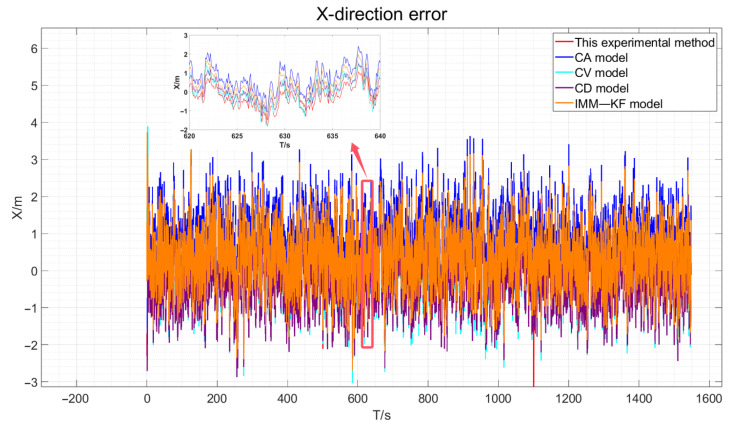
Actual experimental X-direction error and an enlarged view of certain periods.

**Figure 19:**
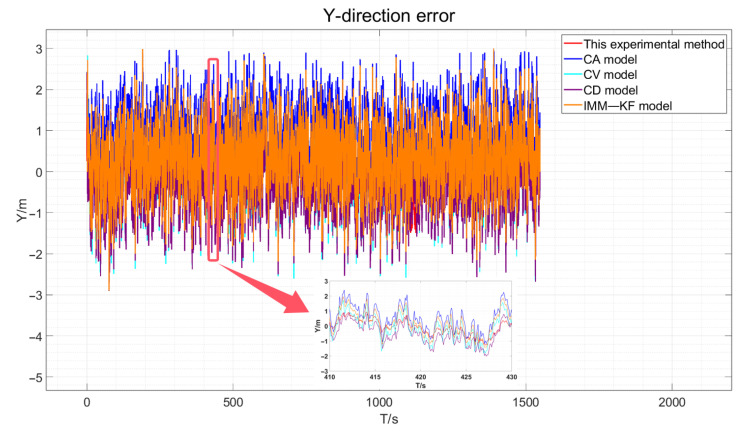
Actual experimental Y-direction error and an enlarged view of certain periods.

**Figure 20:**
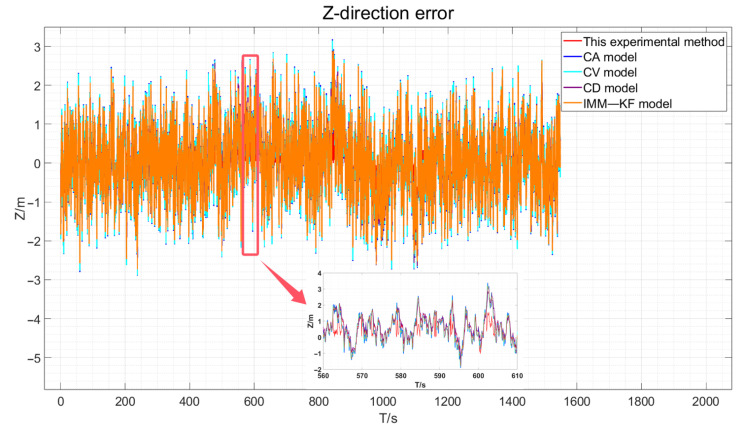
Actual experimental Z-direction error and an enlarged view of certain periods.

The three-direction error results of the actual experiment are drawn, as shown in Figure 18, Figure 19 and Figure 20.

As depicted in Figure 18, Figure 19 and Figure 20, the red line representing the method proposed in this paper is the closest to the true values compared to the other methods. However, due to the presence of various factors affecting positioning in the actual environment, the errors are not very pronounced. Therefore, the data obtained were statistically analyzed. The processed experimental results are quantitatively summarized in Table 5.

**Figure 21:**
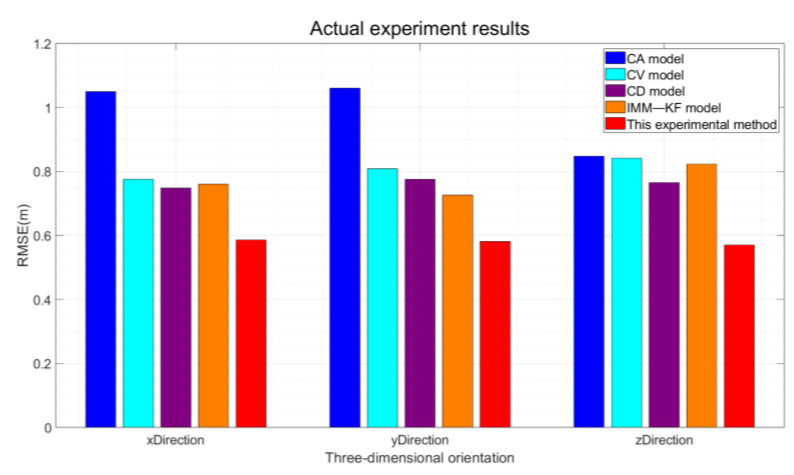
RMSE Statistics in Actual Experiments.

The statistical analysis of the results in Table 5 reveals that the three-direction RMSE derived solely from the least squares results is approximately three meters, with the ME also being notably high. In this experiment, the application of the Kalman filter-based vehicle-mounted GNSS constant-speed model, constant-acceleration model, and constant deceleration model through the establishment of the prediction module and the refinement of the update module effectively reduces the RMSE value and the ME. The implementation of the IMM algorithm, by integrating the constant-speed and constant-acceleration models, achieves lower localization accuracy compared to the traditional constant-speed and constant-acceleration models; however, it still achieves a certain degree of RMSE reduction. The algorithm proposed in this paper leverages data collected from a self-built experimental platform. By discerning different vehicle motion states, flexibly designing matching localization model algorithms, and supplementing the vehicle GNSS localization algorithm with zero-speed constraints upon identifying stationary vehicle states, it surpasses the IMM algorithm in terms of real-time performance, flexible matching, and cost-effectiveness. Compared to the use of constant speed, constant acceleration, and constant deceleration models for enhancing positioning accuracy and against the existing IMM, this paper’s proposed algorithm significantly reduces both the RMSE and ME, thereby demonstrating the effectiveness of the experimental method. The RMSE statistical results are graphically presented in Figure 21.

Specifically, the algorithm proposed in this experiment has achieved significant improvements in positioning accuracy in three dimensions compared to the use of various models. Specifically, it has increased accuracy by 44.1%, 45.4%, and 33.1% compared to the constant acceleration model; by 24.2%, 29.1%, and 32.6% compared to the constant velocity model; by 21.2%, 26%, and 25.5% compared to the constant deceleration model; and by 21.8%, 20.9%, and 31.3% compared to the interactive multi-model approach.

The effectiveness and rationality of the method proposed in this paper are strongly substantiated by the comprehensive results of both the simulation and practical experiments. The commonly employed Kalman filter-based models for vehicle GNSS, including the constant acceleration and constant velocity models, have been shown to significantly enhance navigation and positioning accuracy in both simulated and real-world tests. Moreover, the constant deceleration model also demonstrates a notable improvement in the precision of vehicle navigation and positioning. However, due to their reliance on a single positioning solution model, there remains scope for further improvement in positioning accuracy. The IMM algorithm, proposed as an enhancement, amalgamates the strengths of both models and outputs, resulting in a specific ratio, thereby reducing the RMSE to some extent compared to the individual use of these models.

This experimental approach employs a cost-effective and information-rich fisheye camera as a sensing tool. Establishing an experimental platform captures a vast array of rich image information and discerns the vehicle’s diverse motion states using the inter-frame differential optical flow method. The algorithm presented in this paper showcases excellent real-time capabilities by seamlessly integrating the results of information processing from the camera with the algorithm’s adaptive nature. Notably, given the prevalence of stationary motion states in urban and in-vehicle operational environments, the proposed method improves vehicle navigation and positioning accuracy by incorporating zero-speed constraints following the detection of stationary states. Whether it concerns the reduction of RMSE and average error or the enhancement of positioning accuracy, the method proposed in this experiment exhibits a marked improvement in positioning accuracy compared to the four aforementioned methods, particularly when compared to the IMM algorithm. Consequently, after rigorous validation through simulation and actual experiments, the proposed method demonstrates superior robustness, real-time performance, and flexible matching capabilities, rendering it more effective in achieving accurate in-vehicle GNSS navigation and localization within complex urban environments.

## 5. Conclusions

To address the challenge of current onboard GNSS navigation and positioning models struggling to adaptively align with the rapidly fluctuating vehicle motion states in urban environments, necessitating enhanced positioning accuracy, this paper introduces an elastic filtering algorithm with visual perception for vehicle GNSS navigation and positioning. This innovative algorithm employs a cost-effective skyward fisheye camera that is adept at capturing extensive environmental data around a vehicle’s movement. By leveraging the inter-frame differential optical flow method to discern vehicle motion and rest, it can effectively identify the vehicle’s turning state using the differential speedometer. The algorithm also performs feedback corrections based on the mean value transformation of the optical flow feature points and the general state transitions of the vehicle, such as initiating acceleration post-stationary and decelerating pre-stopping. This enables real-time, accurate judgment of various vehicle motion states and flexible matching with diverse GNSS filtering models. Moreover, given the prevalence of stationary vehicles in urban settings, the algorithm employs a zero-speed constraint to boost the positioning accuracy of in-vehicle navigation during such states, based on the detection of stationary vehicles and adaptive filtering model matching. Following simulation experiments that validated the effectiveness of the proposed algorithm, the method introduced in this paper demonstrated a significant advantage over common algorithms in terms of positioning accuracy during practical experiments. Compared to the IMM approach, the positioning accuracy enhancements in the three dimensions are 21.8%, 20.9%, and 31.3%, respectively.

This paper integrates the environmental information captured by fisheye cameras with the proposed algorithm for elastic binding, which, compared to common and existing methods, offers real-time performance and flexible matching capabilities, resulting in improved positioning accuracy. However, the robustness of the proposed method and the adaptiveness of the judgment thresholds require further enhancement, especially in scenarios such as vehicle deceleration, bumps, and skidding. Additionally, diverse filtering methods and vehicle speeds are factors that affect the positioning accuracy of in-vehicle navigation systems. In future work, it will be necessary to address various scenarios and special conditions of vehicle motion, different speeds, and filtering methods by flexibly employing cutting-edge technologies, such as multimodal combinations, multi-sensor integration, and multi-system fusion.

## Figures and Tables

**Table 1:** Parameters of the experimental equipment.

Equipment Content	Performance Specification and Parameter
DW800 wide-angle fisheye camera	Horizontal field of view of 150° for continuous information acquisition and stable delivery of video images.
Lens center (pixel)	(343, 248)
Focal length (pixel)	218
Image pixel (pixel)	640 × 480
Image acquisition rate (frame)	20 per second

**Table 2:** Vehicle State Determination Result Statistics.

Vehicle Motion States	Stationary	Constant Acceleration	Constant Speed	Constant Deceleration	Turning
Corresponding image epoch (specification: 20 frames per second)	1–2400, 9189–10,533, 14,321–15,482	2401–2680, 10,534–10,766,	2681–3655, 3859–4623, 4913–6464, 6699–7854, 8138–9014, 10,767–11,973, 12,234–13,846	9015–9188, 13,847–14,320	3656–3858, 4624–4912, 6465–6698, 7855–8137, 11,974–12,233

**Table 3:** Simulation Experiment Vehicle Motion Design.

Vehicle Motion States	Stationary	Constant Acceleration	Constant Speed	Constant Deceleration	Turning
Time(s)	1.0–30.0; 197.1–227.0;	31.1–40.0; 118.1–128.0;	40.1–70.0; 79.1–109.0; 128.1–148.0; 167.1–187.0	148.1–158.0; 187.1–197.0	70.1–79.0; 109.1–118.0; 158.1–167.0;

**Table 4:** ME and RMSE in Three Directions from Simulation Experiments.

	ME (m)			RMSE (m)		
	X	Y	Z	X	Y	Z
Least Squares	2.5221	2.3345	2.4798	2.9599	2.9892	3.0211
Constant Acceleration	0.7958	1.3955	0.6645	0.8002	1.4116	0.6403
Constant Velocity	1.0264	1.5787	0.5585	1.1997	1.7803	0.5922
Constant Deceleration	0.9255	1.2564	0.6789	1.0729	1.3155	0.6417
IMM	0.7206	1.0344	0.5498	0.7866	1.0457	0.6183
**This experiment method**	**0.6525**	**0.7041**	**0.5549**	**0.6703**	**0.7764**	**0.5414**

**Table 5:** Actual Experimental Result Statistics.

	ME (m)			RMSE (m)		
	X	Y	Z	X	Y	Z
Least Squares	2.4005	2.3367	2.3788	2.9715	2.9588	2.9899
Constant Acceleration	0.8744	0.8366	0.6784	1.0498	1.0579	0.8496
Constant Velocity	0.6277	0.6200	0.6988	0.7746	0.8154	0.8428
Constant Deceleration	0.7754	0.7156	0.6994	0.7452	0.7819	0.7624
IMM	0.6825	0.6577	0.6274	0.7508	0.7311	0.8267
**This experiment method**	**0.4778**	**0.4356**	**0.4561**	**0.5876**	**0.5787**	**0.5684**

## Data Availability

All original experimental data were collected by the authors and are being used for the first time in this study. To access this data, please contact the corresponding author.
